# Trends in Cervical Cancer Screening Modality in the Active Component U.S. Military, 2013–2023

**Published:** 2025-05-01

**Authors:** Meghan Ginn, Shauna L. Stahlman, Michael T. Fan, Symone Baker Miller

**Affiliations:** Department of Preventive Medicine and Biostatistics, Uniformed Services University of the Health Sciences, Bethesda, MD; Armed Forces Health Surveillance Division, Defense Health Agency, Silver Spring, MD

## Abstract

Cervical cancer screening recommendations have evolved in the past 20 years. Several recent studies have reported on practice pattern changes in the U.S. in response to these guideline changes, but practice patterns have not yet been evaluated in the Military Health System (MHS). Data for active component service women were queried from the Defense Medical Surveillance System for relevant inpatient and outpatient encounter codes within the MHS between January 1, 2013 and December 31, 2023 to identify instances of cervical cancer screening and classify each by modality: cytology alone, HPV alone, and co-testing. Trends in the use of each were evaluated within age categories: younger than age 21 years, ages 21–29 years, ages 30–64 years. A total of 378,952 screening events were captured from 2013 through 2023. MHS practice patterns demonstrated a response to national guideline changes, including increased co-testing and evidence of increasing primary HPV screening among women aged 30–64 years. Cervical cancer screening in women younger than age 21 years markedly decreased following recommendations against screening in this age group. The overall trends in the MHS are similar to those reported in the U.S. general population.

The U.S. has made noteworthy gains in reducing the incidence of cervical cancer, from 9.7 per 100,000 (age-adjusted) in 1999 to 7.4 per 100,000 in 2013. ^1^ New screening technology, namely human papillomavirus (HPV) testing, increased understanding of the pathophysiology of HPV-driven carcinoma, and the introduction of primary prevention through HPV vaccines, continue to drive changes in cervical cancer screening methods and recommendations. ^2^ National 2012 guidelines from both the U.S. Preventive Services Task Force (USPSTF) and the American Cancer Society (ACS) included testing for HPV concurrently with cytology (‘co-test’) for women aged 30-65 years, which allowed safe prolongation of screening intervals from 3 years to 5 years. ^3 , 4^ Primary HPV screening, in which high-risk HPV (hrHPV) is tested first and cytology is performed as a reflex only if HPV is positive, was introduced for 30-65-year olds in 2018 by the USPSTF and expanded to 25-65-year olds by the ACS in 2020. ^2 , 5^ ACS guidelines notably recommended primary HPV as the preferred screening modality, with cytology and co-test acceptable if primary HPV is not available, a stance that has since been put forward for comment by the USPSTF in their draft 2024 cervical cancer screening guidelines. ^2 , 6^ The first patient self-collected vaginal swabs for primary HPV screening were also approved in 2024 by the U.S. Food and Drug Administration, and large multi-site trials are currently underway to evaluate these swabs outside of clinical settings as true ‘at home’ screening tests for cervical cancer. ^7 , 8^

Implementation of new national cervical cancer screening guidelines is a massive undertaking, particularly when those guidelines require new testing modalities. Laboratories often must acquire and validate new equipment, workflows, and testing menus. ^2 , 9 - 11^ Providers must understand, agree with, and offer the new screening modalities to their patients, who then must accept their providers' recommendations. ^9 , 10^ This large expenditure of both human and financial resources may not be immediately feasible. ^2^ Several studies have examined how quickly the updated screening guidelines are implemented, by analyzing trends in use of cervical cancer screening modalities over time, including Qin et al.'s 2021 study of nearly 10 million commercially insured women from 2013 through 2019. ^10 , 11^ Qin et al. found that trends in co-test and cytology screening of 30-65-year olds aligned well with new guidelines but observed minimal uptake of primary HPV screening and some discrepancies in screening modalities of 21-29-year olds. ^11^

**What are the new findings?**

Trends in use of each screening modality—cytology, primary HPV, and co-testing—for active component service women are shifting in response to changing national guidelines. There is emerging evidence of increasing primary HPV screening in women ages 30–64 years, especially after 2021.

**What is the impact on readiness and force health protection?**

Newer cervical cancer screening modalities that incorporate HPV testing, including the possibility of patient self-collected samples, allow for prolonged screening intervals and use in challenging health care settings. Full implementation will likely require significant investment in patient and provider education as well as laboratory processes, with several key questions that remain unanswered.

To our knowledge, these trends have not yet been investigated in the Military Health System (MHS). The MHS experiences the same resource management challenges as the rest of U.S. health care. It must also overcome unique challenges posed by its patient population, some of whom undertake long assignments to remote and austere locations without infrastructure or medical providers necessary to support cytology screenings. ^12^ Applying the same methods of Qin et al. to the MHS, this report aims to reveal trends in cervical cancer screening modalities (primary HPV vs. cytology alone vs. co-test) in active component service members (ACSMs) as a response to changing national guidelines and compare trends to those of the U.S. population previously reported.

## Methods

The Defense Medical Surveillance System (DMSS) was queried for inpatient and outpatient encounters within the MHS between January 1, 2013 and December 31, 2023 that included International Classification of Diseases (ICD), 10th and 9th revisions, Current Procedural Terminology (CPT), and Healthcare Common Procedure Coding System (HCPCS) codes listed in Appendix 2 of Qin et al. Inpatient encounters were included in the query to apply surveillance exclusion criteria (see below), in addition to avoiding unwitting omission of the rare inpatient screening events that met surveillance inclusion criteria. The surveillance period was chosen to capture changes following the 2012 addition of co-testing to national guidelines and include the most current available data.

The surveillance population included all female ACSMs in the Air Force (including Space Force after 2022), Army, Marine Corps, and Navy younger than 65 years, on continuous active component status for the entirety of the calendar year, with routine cervical cancer screening. To identify tests performed for routine cervical cancer screening, as opposed to diagnosis or follow-up to treatment, exclusions were made using ICD-10 / ICD-9 / CPT / HCPCS codes, mirroring those of Qin et al., with the sole addition of CPT code 57530 (trachelectomy). Service women were excluded from analysis if they had documented history of cervical dysplasia or procedure for diagnosis or treatment of dysplasia, such as loop electrosurgical excision and cervical conization, or congenital or acquired absence of uterine cervix.

To maintain comparability with reported U.S. national trends, the modality definitions of Qin et al. were also utilized. Cervical cancer screening was classified as ‘co-test’ if DMSS documentation included both cervical cytology within the calendar year and hrHPV testing within 3 days preceding or 30 days after cytology. Screening was classified as ‘cytology alone’ if cervical cytology was performed within the calendar year but no hrHPV was documented within the guidelines of ‘co-testing’. Finally, screening was classified as ‘HPV alone’ if at least 1 hrHPV test was documented in the calendar year but no cervical cytology was performed. The screening test categories were mutually exclusive, with individuals counted only once per year, with prioritization for co-testing, followed by cytology alone and HPV alone. Individuals were further classified by demographics and age as recorded on the date of cervical cancer screening. Descriptive statistics were used to evaluate trends in use of each cervical screening modality over the duration of the surveillance period by age category, reported as a percentage of women within the surveillance population screened by each modality each year. Over-all screening compliance (i.e., percentage of women ‘up to date’ at any given time) was not considered as an outcome.

## Results

After exclusions, the surveillance population averaged 147,476 women in service each year, ranging from 123,828 in 2013 to 168,444 in 2021 **(Tables 1** and **2)** . Distributions of age, race and ethnicity, branch of military service, and military rank within the surveillance population were consistent throughout the surveillance period. The largest proportions of the population were 21-29 years of age (59.5%), non-Hispanic White (42.7%), and enlisted (45.8%). Service distribution was nearly equal among the Army, Air Force, and Navy, with a much smaller proportion of Marine Corps members.

**TABLE 1.** Study Population Demographics, Annual Average, 2013–2023

	Average	
	No.	%
Age, y		
<21	15,094	10.2
21–24	46,487	31.5
25–29	41,308	28.0
30–39	34,740	23.6
40–49	8,584	5.8
50–64	1,264	0.9
Service branch		
Army	48,563	32.9
Navy	44,949	30.5
Air Force, Space Force	43,038	29.2
Marine Corps	10,927	7.4
Race and ethnicity		
White, non-Hispanic	62,909	42.7
Black, non-Hispanic	35,587	24.1
Hispanic	27,779	18.8
Other	18,497	12.5
Unknown	2,705	1.8
Rank, grade		
Junior enlisted (E1–E4)	67,480	45.8
Senior enlisted (E5–E9)	51,289	34.8
Warrant officer (W)	862	0.6
Junior officer (O1–O3)	19,564	13.3
Senior officer (O4–O10)	8,281	5.6

Abbreviations: y, years; E, enlisted; W, warrant officer; O, officer.

**TABLE 2.** Testing Modality, Year of Surveillance and Age Group, Absolute Number and Relative Percent, 2013–2023

Year		2013		2014		2015		2016		2017		2018	
Population total		123,828		127,446		130,187		133,938		137,796		147,760	
Age group, y	Screening modality	No.	%	No.	%	No.	%	No.	%	No.	%	No.	%
<21	Total, age group	12,130		13,080		12,865		13,858		14,965		16,882	
	Co-test	6	0.1	4	0.0	3	0.0	6	0.0	9	0.1	7	0.0
	Cyto	371	3.1	307	2.4	214	1.7	210	1.5	201	1.3	206	1.2
	HPV	15	0.1	9	0.1	7	0.1	20	0.1	32	0.2	24	0.1
21–29	Total, age group	70,384		74,123		77,701		80,714		83,350		89,381	
	Co-test	115	0.2	112	0.2	216	0.3	468	0.6	692	0.8	1,056	1.2
	Cyto	19,659	27.9	20,268	27.3	21,553	27.7	23,280	28.8	23,458	28.1	24,333	27.2
	HPV	74	0.1	52	0.1	96	0.1	94	0.1	158	0.2	123	0.1
30–64	Total, age group	41,314		40,243		39,621		39,366		39,481		41,497	
	Co-test	808	2.0	1,015	2.5	1,195	3.0	1,628	4.1	2,014	5.1	2,364	5.7
	Cyto	9,542	23.1	9,417	23.4	8,725	22.0	7,934	20.2	7,007	17.7	6,685	16.1
	HPV	114	0.3	118	0.3	140	0.4	154	0.4	190	0.5	210	0.5

Year		2019		2020		2021		2022		2023	
Population total		155,605		164,708		168,444		166,864		165,661	
Age group, y	Screening modality	No.	%	No.	%	No.	%	No.	%	No.	%
<21	Total, age group	17,782		18,550		17,245		14,726		13,946	
	Co-test	9	0.1	4	0.0	10	0.1	4	0.0	7	0.1
	Cyto	160	0.9	150	0.8	164	1.0	120	0.8	73	0.5
	HPV	10	0.1	7	0.0	5	0.0	9	0.1	7	0.1
21–29	Total, age group	93,884		98,650		101,170		99,844		96,543	
	Co-test	1,283	1.4	1,437	1.5	1,067	1.1	1,221	1.2	1,458	1.5
	Cyto	26,399	28.1	24,195	24.5	26,811	26.5	23,042	23.1	20,489	21.2
	HPV	161	0.2	192	0.2	227	0.2	339	0.3	464	0.5
30–64	Total, age group	43,939		47,508		50,029		52,294		55,172	
	Co-test	2,920	6.6	2,708	5.7	3,241	6.5	3,460	6.6	3,999	7.2
	Cyto	6,988	15.9	6,459	13.6	7,581	15.2	6,525	12.5	5,762	10.4
	HPV	281	0.6	351	0.7	439	0.9	784	1.5	1,212	2.2

Abbreviations: y, years; Cyto, cytology; HPV, human papillomavirus.

**FIGURE 1.**
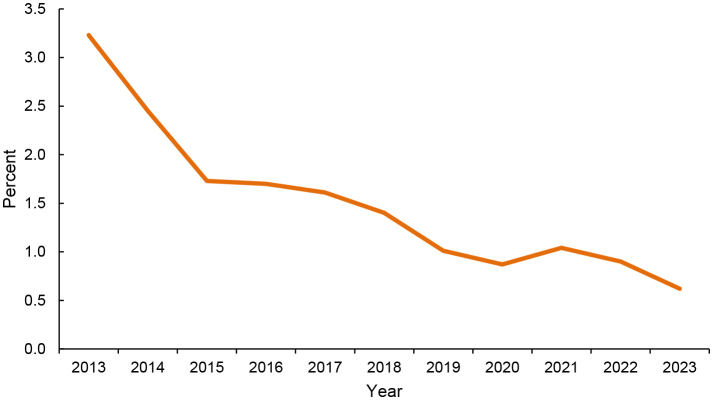
Percentage of Women Younger than Age 21 Years with Screening for Cervical Cancer, Any Modality, 2013–2023

**FIGURE 2.**
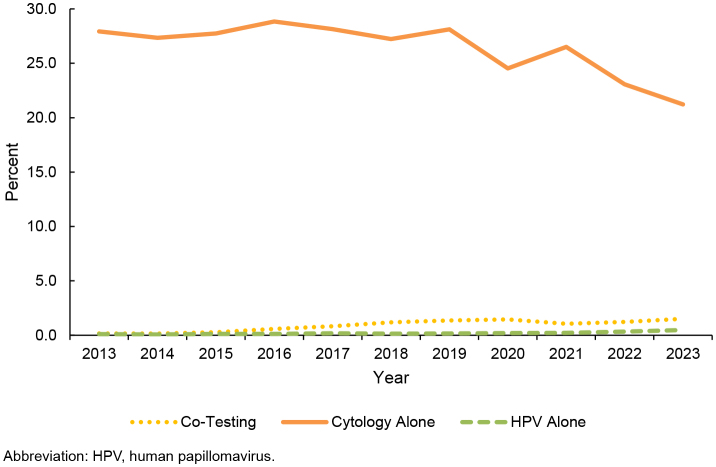
Percentage Use of Each Screening Modality for Cervical Cancer, Women Ages 21-29 Years, 2013–2023

**FIGURE 3.**
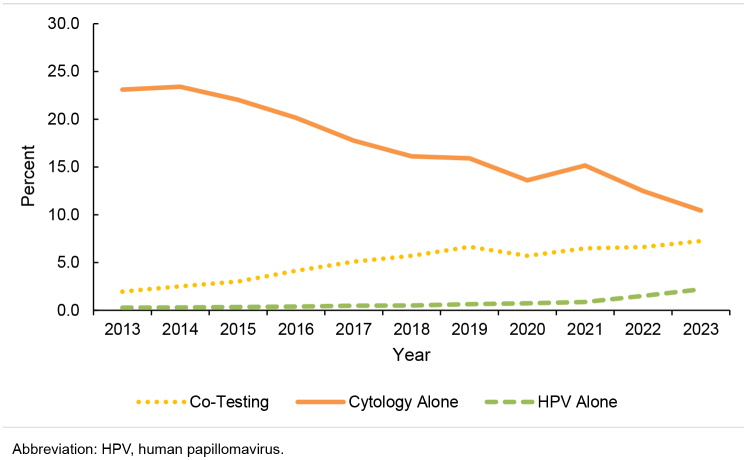
Percentage Use of Each Screening Modality for Cervical Cancer, Women Ages 30-64 Years, 2013–2023

A total of 378,952 cervical cancer screenings were captured during the surveillance period, classified according to the methods described. An average of 34,450 screenings occurred each calendar year, ranging from 30,704 in 2013 to 39,545 in 2021 **(Table 2)** . Cervical cancer screening in individuals younger than age 21 years declined steadily during the surveillance period, from 3.2% in 2013 to 0.6% in 2023 **(Figure 1)** . The majority (83.1–95.9%) of screenings in the younger than age 21 group were classified as cytology. Among 21-29 year olds, use of cytology alone remained steady throughout most of the surveillance period (27.9% in 2013, 28.1% in 2019) but decreased after 2019, to 21.2% in 2023 **(Figure 2)** . Co-test and HPV alone in the 21-29-year age group increased during the surveillance period, but use of both remained low: In 2019, 1.5% of women ages 21-29 were screened by co-test, 0.5% were screened by HPV alone **(Figure 2)** . Similar, but more pronounced, trends were observed in the 30-64-year age group **(Figure 3)** . Use of cytology alone decreased by more than 50% (23.1% in 2013 to 10.4% in 2023) among 30-64 year olds, while co-test more than tripled over the surveillance period (2.0% to 7.2%). HPV alone also increased in this age group, with the most marked increase occurring after 2021 (0.3% in 2013, 0.9% in 2021, 2.2% in 2023) **(Table 2** , **Figure 3)** .

## Discussion

Understanding how practice patterns within the MHS change in response to guideline shifts is important because it reflects a myriad of factors influencing those changes such as provider education, patient and provider preference, and testing infrastructure, which may then inform how well the MHS is positioned to respond to future changes in screening guidelines. These results show that trends in the MHS are overall as expected in response to national guideline changes, and similar to trends in the U.S. reported by Qin et al. in 2021. Cervical cancer screening guidelines have recommended against screening women younger than age 21 years since 2012. ^4^ Screening in this age group declined precipitously in the MHS, to 1.0% in 2019, well below the 9.0% reported in 2019 among women ages 18-20 years in the U.S. population. ^11^ Similarly, screening modalities in women ages 30-64 years shifted in both the MHS and U.S. populations in response to the 2012 guideline updates, increasingly favoring co-testing as opposed to cytology alone. Interestingly, while use of cytology alone in 30-64 year old ACSMs declined throughout the surveillance period, it remained the more common modality over co-testing. In contrast, Qin et al. reported that co-testing overtook cytology alone between 2014 and 2015. ^11^ Whether this represents a difference in testing characteristics, including rates of squamous atypia or length of time between reporting of cytology and hrHPV results, or a true preference for cytology and reflex HPV over co-testing within the MHS, is unclear.

Qin et al., among others, have reported a decline in use of cytology for cervical cancer screening in individuals 21-29 years of age. Potential explanations have included genuinely decreased screening among recipients of the HPV vaccine who are now entering the screening pool, as well as an artificial decline in apparent use as a result of extended screening intervals from annually to every 3 years. ^11^ Among ACSMs, cervical cancer screening is required as part of individual medical readiness regardless of HPV vaccination status, making this an unlikely explanation for the decrease in cytology after 2020. The 2019 management guidelines of the American Society for Colposcopy and Cervical Pathology differ between patients younger than age 25 years and those aged 25-29 years with atypical squamous cells of undetermined significance (ASCUS) on cytology screening, with preference to HPV testing for further risk stratification in those aged 25-29 years. ^13^ Data on the results of cervical cancer screening were not included in this analysis, and it is conceivable that a rise in ASCUS results among those 25-29 years of age caused an artificial decline in cytology percentages as ASCUS cytology results triggered reflex HPV and subsequent misclassification as ‘co-test’.

This study has several limitations. As mentioned, the definitions of screening modality (co-test vs. cytology alone vs. HPV) were chosen to correlate with Qin et al., for comparison to national trends. There is likely misclassification among modalities, however, including abnormal cytology with reflex HPV misclassified as co-test, positive primary HPV with reflex cytology misclassified as co-test or cytology (depending on timing of cytology result), and co-test misclassified as cytology (if HPV is coded significantly before cytology result). Pathology data were not available at the time of this analysis, which precluded use of laboratory-generated data to validate or quantify degree of possible misclassification by medical encounter data. The surveillance period of this study overlaps with the COVID-19 pandemic, which may have affected screening rates during the pandemic years but would have unlikely influenced testing modality. Finally, the analysis was intended only for women undergoing routine screening, but women with a history of dysplasia may have been mistakenly included if that history was not documented within the electronic health record.

Future work should aim to confirm the trends reported here with validation by laboratory data. The performance characteristics of primary HPV screening in the ACSM population should be evaluated, including its cost effectiveness and negative predictive value in a population with a potentially higher rate of HPV infection. ^12^ Paradigm shifts in large-scale screening programs are gradual, and continued surveillance should be considered for further evaluation and guidance of the process as practice patterns and guidelines continue to evolve.
